# Rapid storage and retrieval of genomic intervals from a relational database system using nested containment lists

**DOI:** 10.1093/database/bat056

**Published:** 2013-07-26

**Authors:** Laura K. Wiley, R. Michael Sivley, William S. Bush

**Affiliations:** ^1^Department of Biomedical Informatics, Center for Human Genetics Research, Vanderbilt University, 2215 Garland Ave, Nashville, TN 37232 USA and ^2^Department of Electrical Engineering and Computer Science, Center for Human Genetics Research, Vanderbilt University, 2215 Garland Ave, Nashville, TN 37203 USA

## Abstract

Efficient storage and retrieval of genomic annotations based on range intervals is necessary, given the amount of data produced by next-generation sequencing studies. The indexing strategies of relational database systems (such as MySQL) greatly inhibit their use in genomic annotation tasks. This has led to the development of stand-alone applications that are dependent on flat-file libraries. In this work, we introduce MyNCList, an implementation of the NCList data structure within a MySQL database. MyNCList enables the storage, update and rapid retrieval of genomic annotations from the convenience of a relational database system. Range-based annotations of 1 million variants are retrieved in under a minute, making this approach feasible for whole-genome annotation tasks.

**Database URL:** https://github.com/bushlab/mynclist

## Introduction

A typical genomic annotation is represented in the form of an interval (i.e. a range of base-pair positions), such as the boundaries of a gene. Millions of interval-based genomic annotations are available from multiple online resources, most notably the UCSC genome browser, which provides genomic elements in BED file format (chromosome, start, stop and label). Although this format is convenient for browsing a single genomic region or base-pair position through an online genome browser, large collections of genomic intervals are difficult to rapidly search. A common task in genomics is to search interval data (such as a collection of BED files) to identify a set of interval-based annotations that overlap with a target interval. For example, a user may want to identify genomic elements located within a deleted region. The main challenge for these types of interval-based queries is maintaining a sorted order of intervals to facilitate rapid searches—a task known as *indexing* within database management systems (DBMS).

**Figure 1.**
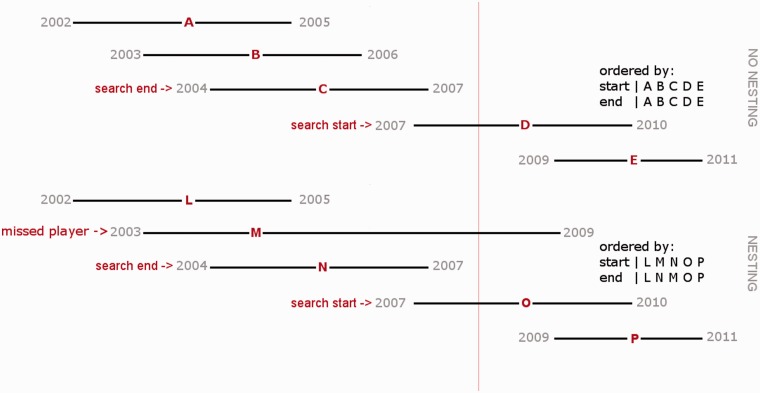
Nested intervals disrupt ordering and query strategies. Given these two historic rosters and the question: who played in 2008 (represented with the red line)? In the non-nested example, sorting individuals by their first-year results was done in the same order as when we sort by the players’ last year. Thus, we can use a traditional index on start and end positions to quickly scan backwards, stopping at the 2004–2007 range (player C). In the nested example, the ordering of players by their last year on the team is different from when we sort by their first year. Thus, if we implement the same reverse search technique used in the non-nested example, stopping our query at the 2004–2007 range (player N) would skip player M. Therefore, we must search the entire set of players when intervals are nested.

Indexing is complicated by nested intervals, in which one interval occurs entirely within the boundaries of a second interval. If no nested intervals are present, sorting intervals by their start position also properly sorts their end positions. Relational database indexing strategies, which assume a single key, can thus properly order non-nested intervals. However, when a nested interval occurs, sorting based on start position no longer guarantees that the end positions will be properly ordered, as shown in Figure 1. To better understand the consequences of this problem, consider the more familiar task of sorting date intervals. Consider a football team that each year accepts some new players and loses some existing players. Some players may stay for only a short time, whereas others spend their entire careers with the same team. As such, one player’s career with that team may be entirely nested within another’s, illustrated in Figure 1. Now consider the question: who played for this team in year 2008? If player careers were non-nested and ordered (as for players A–E), we could identify the last player to join in 2008, and then work our way backward through the sorted list until we found the first player (C) who quit before 2008. However, consider a second set of players (L–P), if the team membership of some players is nested within the membership of other players, there could be a player (M) who joined before player N but stayed with the team after 2008. Because we can draw no conclusions about the ordering of when players quit, we must scan *all players* who joined before year 2008. In the worst case, we ask about the current year, requiring us to scan everyone who ever played for the team.

In aggregate, table scans like these become very slow, reducing the feasibility of relational databases for genomic annotation tasks (1). An interval-based data structure called Nested Containment List (NCList) was developed to address this issue (2). The NCList data structure and associated algorithms were released as part of a Python graph database library, *pygr*. This implementation achieved 5–500-fold faster query times than other DBMS-style indexing methods available at the time. Since publication, the NCList structure has been used for sequence alignment using UCSC genome alignments (3, 4), processing ChIP-seq and ChIP-chip data (5), and has been incorporated into the popular JBrowse genome browser (6).

The NCList data structure achieves better query performance by hierarchically organizing all nested intervals. This guarantees that each search space consists of contiguous non-nested intervals (i.e. the search space is ordered both on start and stop). Each interval points to a sublist of all completely contained intervals. The query algorithm follows a recursive path that returns all overlapping intervals within all contained sublists. The time complexity of this type of query is O(MlogN), where M is the depth of the tree and N is the database size.

Existing NCList implementations provide exceptional performance for interval query operations but lack the flexibility and convenient interface of a traditional DBMS, most notably the ability to perform in-place updates on an existing structure. MyNCList is an implementation of the NCList data structure in the common database management software MySQL. It provides mechanisms for in-place updates and querying using MySQL stored procedures. These tools facilitate the construction of a single updatable repository of information for the rapid annotation of genomic sequence variants.

## Implementation

Construction of the NCList data structure consists of a Python script that accepts interval annotations in the BED file format. Intervals are processed and placed into a nested containment tree. After tree generation, the script prepares the MySQL data structure and stores the data into node, edge and masterkey tables. The masterkey table uses a numerical identifier to link each interval stored in the node table to its alphanumeric label from the original BED file. Because the intervals in the node table are hierarchical, the edge table is used to link parent intervals to their nested child intervals. The node table contains the id, start and stop positions of all intervals in the structure and a sublist (sub) value. All intervals contained by the same parent are assigned the same sub value, thus grouping intervals in a hierarchical relationship. The base intervals (i.e. those intervals that do not fit within any other interval) are assigned to sub 0, the root of the tree. When a range may fit within multiple partially overlapping intervals, the child interval is assigned based on a user-specified parameter; the default is to assign the interval to the first parent in the sorted order. The tree construction algorithm begins by sorting all intervals by their start positions. Construction of the nested containment tree is performed in a single pass.

In-place updates are accomplished using two stored procedures (addition and removal of intervals) in MySQL. To update the coordinates of an interval, it must be removed and reinserted with the new start and stop positions. To increase the speed of these procedures, we create two binary tree indices on the node table; one on the sub, start and stop position of each range, and one on the start and stop values only. The edge table is indexed by interval id and the sub values.

**Figure 2.**
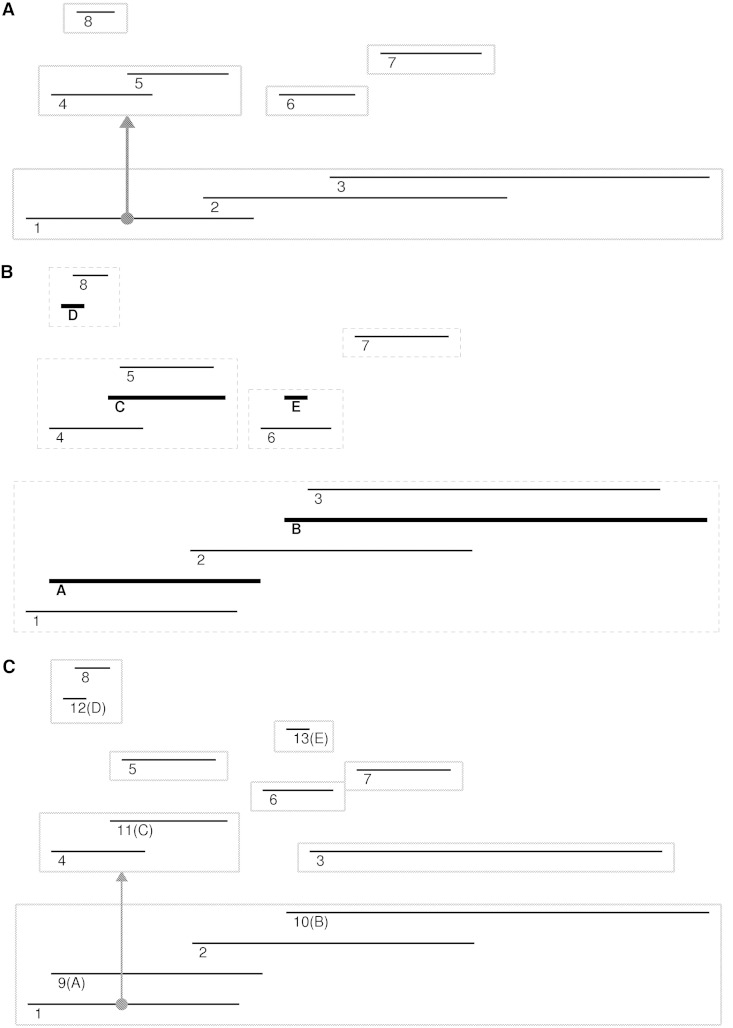
The NCList algorithm and update procedure. (**A**) The original interval organization where contiguous overlapping intervals are grouped and individual intervals point to sublists containing the completely overlapped intervals. (**B**) Transition structure highlighting the intervals to be added (bolded with alphabetic labels). (**C**) The completed structure with inserted intervals fully incorporated.

There are multiple distinct conditions possible when adding or removing ranges from the structure (shown as the alphabetic ranges in Figure 2). Intervals in the bottom layer of the structure (intervals A and B) are automatically assigned to sub 0, as they are not completely overlapped by any other interval. Interval A contains no sub 0 ranges, so it is simply added to the node table, and no other alteration is necessary. However, interval B does contain a sub 0 interval (interval 3). Thus, interval 3 is given a new sub value, and an entry in the edge table is added pointing from interval B to this new value. Intervals C, D and E are found deeper in the structure. When an interval is completely contained and contains children (e.g. interval C), the inserted interval (C) is given the sub value of its child (interval 5). The children are assigned a new sub value, and an entry in the edge table pointing to the new sub is added. When there are no child intervals, additions are less complex. If the parent of an added interval (interval D) already has children, the interval is simply added to the node table with the same sub value as the other children. If the interval does not have any siblings (interval E), a new sub value is assigned, and an entry into the edge table pointing from the parent is added.

The deletion mechanism is less complex, as two interval types (A and D) can be deleted without any alterations. Interval E requires the entry in the edge table pointing from its parent be removed. The children of interval B are reassigned to sub 0 and the entry in the edge table removed. Interval C is similar, here the children are assigned the sub value of interval C, and the entry in the edge table is removed.

## Results and discussion

We evaluated the performance of the MyNCList process using a combined set of multiple gene exons with ∼973 000 intervals. Construction of the Nested Containment Graph completes within seconds, but does require the BED file to be entirely loaded into memory. The insert mechanism for this database adds ∼400 intervals per minute, whereas the delete mechanism removes ∼2800 intervals per minute. The scalability of these operations is typical of other tree-based data structures.

**Figure 3.**
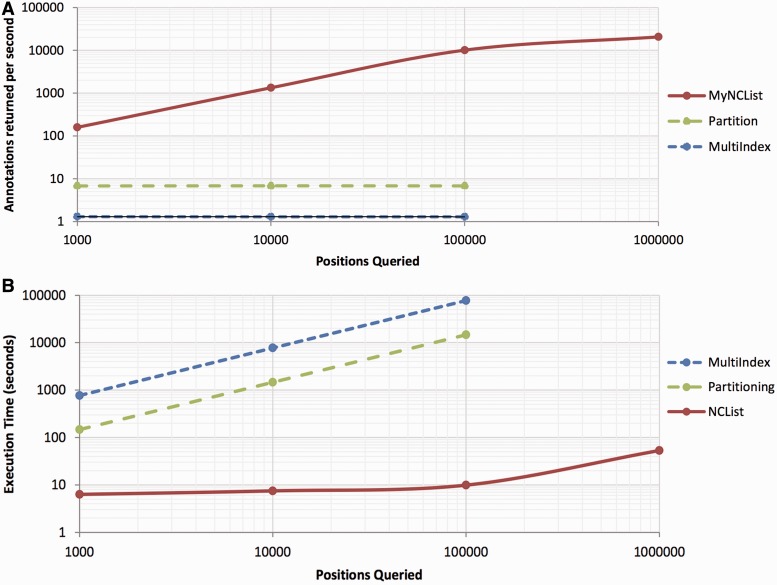
Query performance of MyNCList compared with partition-based and multiple-indexing strategies in MySQL. Performance is shown in annotations per second (**A**) and in raw execution time (**B**).

We compared the query performance of MyNCList with two other popular indexing strategies; a partition-based index and a start/stop position multi-index. We generated tables containing random sets of single base-pair positions in increasing orders of magnitude (1000, 10 000, 100 000 and 1 000 000) drawn uniformly across the human genome to simulate variable sequence positions. We then annotated these positions using a test database derived from exons extracted from the Ensembl, Aceview and UCSC genome databases, equaling ∼973 000 intervals, reflective of a true annotation task. Simulated variant positions were then joined to the test database using the multi-index strategy, using the partitioning strategy and using the MyNCList stored procedure. All database operations were performed with MySQL query and key cache features disabled. The performance of these methods for our test database is shown in Figure 3. Notably, annotation time for the indexed tables scales linearly with the number of locations, whereas annotation time for MyNCList scales logarithmically. As such, a query of 100 000 positions completes in 9.8 s using MyNCList, but requires several hours to complete with other indexing strategies. A table of 4.7 million positions is annotated in 3.1 min using MyNCList, exceeding the performance of the ANNOVAR, making this a viable method for high-throughput annotation of variants discovered from whole-genome sequence data (1). We also expect these measures to be conservative, as optimization of the MySQL key cache and other features will improve performance.

## Availability

The Python script and MySQL package are available for non-commercial research institutions. For full details see http://chgr.mc.vanderbilt.edu/bushlab/.
